# The Dundee Resource for Sequence Analysis and Structure Prediction

**DOI:** 10.1002/pro.3783

**Published:** 2019-11-28

**Authors:** Stuart A. MacGowan, Fábio Madeira, Thiago Britto‐Borges, Mateusz Warowny, Alexey Drozdetskiy, James B. Procter, Geoffrey J. Barton

**Affiliations:** ^1^ Division of Computational Biology College of Life Sciences, University of Dundee UK

## Abstract

The Dundee Resource for Sequence Analysis and Structure Prediction (DRSASP; http://www.compbio.dundee.ac.uk/drsasp.html) is a collection of web services provided by the Barton Group at the University of Dundee. DRSASP's flagship services are the JPred4 webserver for secondary structure and solvent accessibility prediction and the JABAWS 2.2 webserver for multiple sequence alignment, disorder prediction, amino acid conservation calculations, and specificity‐determining site prediction. DRSASP resources are available through conventional web interfaces and APIs but are also integrated into the Jalview sequence analysis workbench, which enables the composition of multitool interactive workflows. Other existing Barton Group tools are being brought under the banner of DRSASP, including NoD (Nucleolar localization sequence detector) and 14‐3‐3‐Pred. New resources are being developed that enable the analysis of population genetic data in evolutionary and 3D structural contexts. Existing resources are actively developed to exploit new technologies and maintain parity with evolving web standards. DRSASP provides substantial computational resources for public use, and since 2016 DRSASP services have completed over 1.5 million jobs.

## 1 INTRODUCTION

The flood of sequence data across all species continues to grow in rate and volume. While there are many challenges in managing these large datasets, the major hurdle is to use the raw sequence data to inform our knowledge and understanding of biological systems. In order to achieve this goal, accurate and reliable software tools are required to make structural and functional predictions from the sequence data. Over 30 years, our group has developed innovative software packages, web servers, and databases that allow the structure and function of protein sequences to be probed and has used these in conjunction with experiments to improve understanding of specific biological systems.

**Figure 1:**
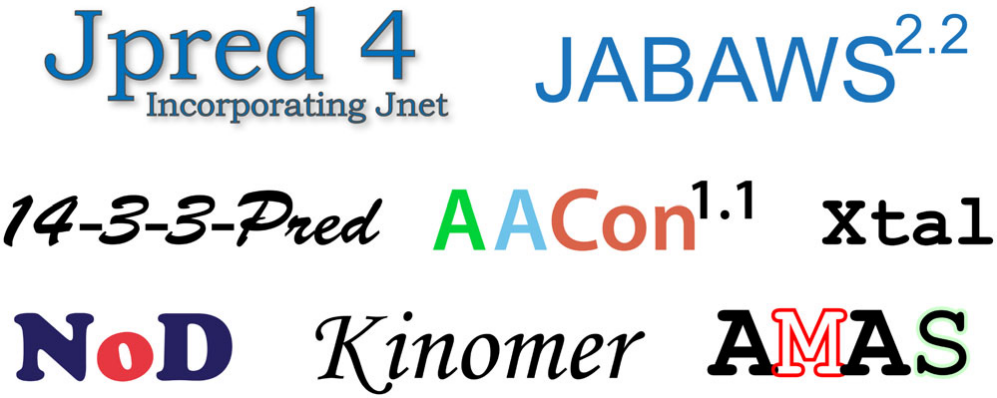
The Dundee Resource for Sequence Analysis and Structure Prediction

The Dundee Resource for Sequence Analysis and Structure Prediction (DRSASP; Figure 1) encapsulates many of these methods alongside techniques developed by other groups as a collection of publicly available protein sequence analysis web services. The resource provides convenient access through websites, application programming interfaces (APIs), and the Jalview^1^ analysis workbench to a variety of algorithms including secondary structure prediction, disorder prediction, multiple sequence alignment, evolutionary conservation calculations, and other functional site predictions.^2^, ^3^, ^4^, ^5^, ^6^, ^7^, ^8^, ^9^, ^10^ DRSASP helps to translate Barton Group research into new web services accessible to a wide community as well as ensuring the sustainability of the popular JPred^2^ and JABAWS.^10^ Initially, DRSASP comprised JPred3,^11^ JABAWS:MSA,^12^ and Kinomer.^5^ Over the last few years, new services have been added such as NoD^9^ and 14‐3‐3 Pred,^3^ and our main services have undergone significant updates. The sustained contribution and relevance of DRSASP has been recognized in the granting of Elixir‐UK Tier 1 Resource status.^13^ This signifies Elixir‐UK's view that DRSASP is an important contributor in the strategic area of Protein Structure and Function. In this article, we summarize the current DRSASP (August 2019) and look forward to new resources that will be added in the near future.

## 2 THE DRSASP TOOLBOX

Table 1 presents an overview of the DRSASP tools and categorizes their application, availability, and technology. The tools address a range of general biological questions: What is the structure of the protein? Will the protein crystallize? Which amino acid residues are conserved across a set of homologues and what type of conservation is present (e.g., identity, hydrophobicity, charge)? Which residues are important for functional specificity? Where does the protein localize in the cell? Are any residues likely to be involved in protein–protein interactions? In terms of technology, 14‐3‐3‐Pred, NoD, and the XTal suite are implemented by sequence‐trained machine learning algorithms; Kinomer is a profile HMM (Hidden Markov model)‐based method; JPred is a multiple neural network method trained from sequence alignment profiles, and AACon and Analysis of Multiply Aligned Sequences (AMAS)^14^ contain a variety of residue set‐based calculations. JABAWS itself is a web service framework with which DRSASP serves a range of sequence analysis methods. Most DRSASP services are accessible via web forms, which are mainly suitable for small‐scale analyses. For bulk analyses, some services provide programmatic‐APIs and/or precomputed datasets. Many services are available directly from Jalview^1^ or provide results in Jalview compatible format. In the following sections, we provide a concise description of each tool covering what it does, how it works, how it can be applied through research examples, and how it is used.

**Table 1:** Summary of the DRSASP tools

			Availability^a^					
Service^b^	Application	Tech	HTML	API	Jalview^1^	Dataset	Released	References
JPred4	Protein 2° structure and solvent accessibility prediction	ANN	✓	✓	✓	✓^c^	2015	2
JABAWS 2.2	Bioinformatics tool web services framework. Provides: MSA, conservation analysis, disorder prediction and RNA 2° structure prediction	Multiple		✓	✓		2018	10
Slivka^d^	Successor to JABAWS (see above)		(✓)	(✓)	(✓)		—	
ProteoCache^d^	DRSASP data warehouse			(✓)		(✓)	—	
pyDRSASP^d^ (ProteoFAV) (ProIntVar) (VarAlign)	Variant and structure analyses			(✓)		(✓)	—	
AACon	Conservation			✓	✓			
14‐3‐3‐Pred	PPIs	ANN SVM PSSM	✓	✓	✓^e^		2015	3
NoD	Subcellular localization	ANN	✓			✓	2011	9
Kinomer	Sequence classification	pHMM	✓			✓	2009	5
XTal (OB‐score) (XANNPred) (ParCrys)	Crystallization propensity, Construct design	Z‐score, ANN, Parzen window	✓			✓^f^	2008	6, 7, 8
AMAS	Functional residues		✓				1993	14

*Note*: Columns—“Service”: The name of the DRSASP service; “Application”: The application area; “Tech”: implementation technology—ANN (Artificial Neural Network—machine learning), SVM (Support Vector Machine), pHMM (Profile Hidden Markov Model), PSSM (Position Specific Scoring Matrix). “HTML”: Tick means service has a web page form interface; “API”: Indicates the service has an Application Programming Interface; “Jalview”: shows services that are directly accessible from Jalview. “Dataset”: Indicates availability of datasets associated with the method. “Released”: First release date of the service.

Abbreviation: AMAS, Analysis of Multiply Aligned Sequences.

a Parentheses denote that the availability is in development.

b Parentheses denote subcomponents of a service.

c The JPred training and test datasets are available for download from the website. Access to precomputed JPred predictions for certain proteomes (e.g., Human) will be available from ProteoCache (§3.2) in future but for now are obtainable from the API or upon request from the authors.

d Service is in development.

e 14‐3‐3‐Pred provides Jalview compatible feature files.

f OB‐Score predictions are available for Pfam 31.0.

### 2.1 *JABAWS: Java bioinformatics analysis web services*

One of our objectives for DRSASP is to deliver resources via a common interface and to make it easy for others to deploy the same services on their own computing infrastructure. With this in mind, we developed the JABAWS^10^, ^12^ framework. JABAWS simplifies the provision of bioinformatics tools as web services by abstracting web interfaces, tool wrapping, wrapper execution, and data models. The DRSASP instance of JABAWS provides access to multiple sequence alignment methods, disorder predictors, an RNA secondary structure predictor, and methods for conservation calculation from multiple sequence alignments.

For multiple sequence alignment, JABAWS includes Clustal Omega,^15^ Clustal W,^16^ Mafft,^17^, ^18^ Muscle,^19^ T‐coffee,^20^ Probcons,^21^ MSAProbs,^22^ and GLProbs.^23^ The availability of these varied multiple sequence alignment programs allows the user to select the best tool for the sequences they wish to align or to compare the results from different algorithms interactively in Jalview or programmatically using the JABAWS client. This approach can also be taken with the multiple options JABAWS provides for residue conservation scoring and disorder prediction. For disorder prediction, we have DisEMBL,^24^ IUPred,^25^ Jronn,^26^ and GlobPlot,^27^ and there are examples where users report the results from two or more of these options.^28^ For MSA interpretation, 17 conservation scores and the SMERFS score^4^ for functional site prediction are available through JABAWS, implemented with our AACon software discussed further in §2.3. For RNA secondary structure prediction, JABAWS provides the RNAalifold method from the ViennaRNA package.^29^

JABAWS allows the specification of command‐line parameter presets. For example, in addition to the default settings, MUSCLE^19^ is configured with separate presets that are suitable for protein alignments and nucleotide alignments whilst MAFFT^18^ presets are configured to implement the NW‐NS‐PartTree‐1, FFT‐NS‐i, FFT‐NS‐1, L‐INS‐i, E‐INS‐i, and G‐INS‐i strategies. For maximum flexibility, command‐line options are exposed via the JABAWS interface allowing users to run tools with options suitable for their own needs.

Most Jalview^1^ users will access the Dundee JABAWS instance as this is preconfigured by default Jalview installations. This makes JABAWS functions accessible immediately after installing Jalview. If a user prefers to keep their data local, work without access to the internet, or tackle very large problems, they may wish to install JABAWS on their personal computer or site‐wide computing resource at their institution. The simplest way to create a JABAWS instance is with the JABAWS virtual appliance or Docker container (see http://www.compbio.dundee.ac.uk/jabaws22/archive/docker/Dockerfile), but a WAR file (Web Application Archive) is provided that is better suited for institutional installations. Jalview can be configured to use the alternative JABAWS instance via *Tools* → *Preferences* → *Web Services*. JABAWS services can also be accessed programmatically via a downloadable command‐line client. Alternatively, users may interface with the JABAWS SOAP API with their own preferred SOAP client. These modes are best suited to users who wish to use JABAWS service for high‐throughput analyses or as part of computational pipelines. The public JABAWS service at http://www.compbio.dundee.ac.uk/JABAWS/ currently has no fair usage policies imposed, but public jobs are restricted to defined maxima for the number of submitted sequences and average sequence length. These restrictions are applied on a tool/preset specific basis and are obtained via SOAP operations, for example, with the *limits* argument to the JABAWS command‐line client. Limits vary from 500–2,000 sequences for sequence alignment, 2,000–5,000 sequences for disorder prediction, and 2,000–10,000 sequences for disorder calculations. Additionally, all jobs are limited to 1 h of compute time. Jobs larger than the relevant size limits will not be accepted, and long running jobs are terminated.

**Figure 2:**
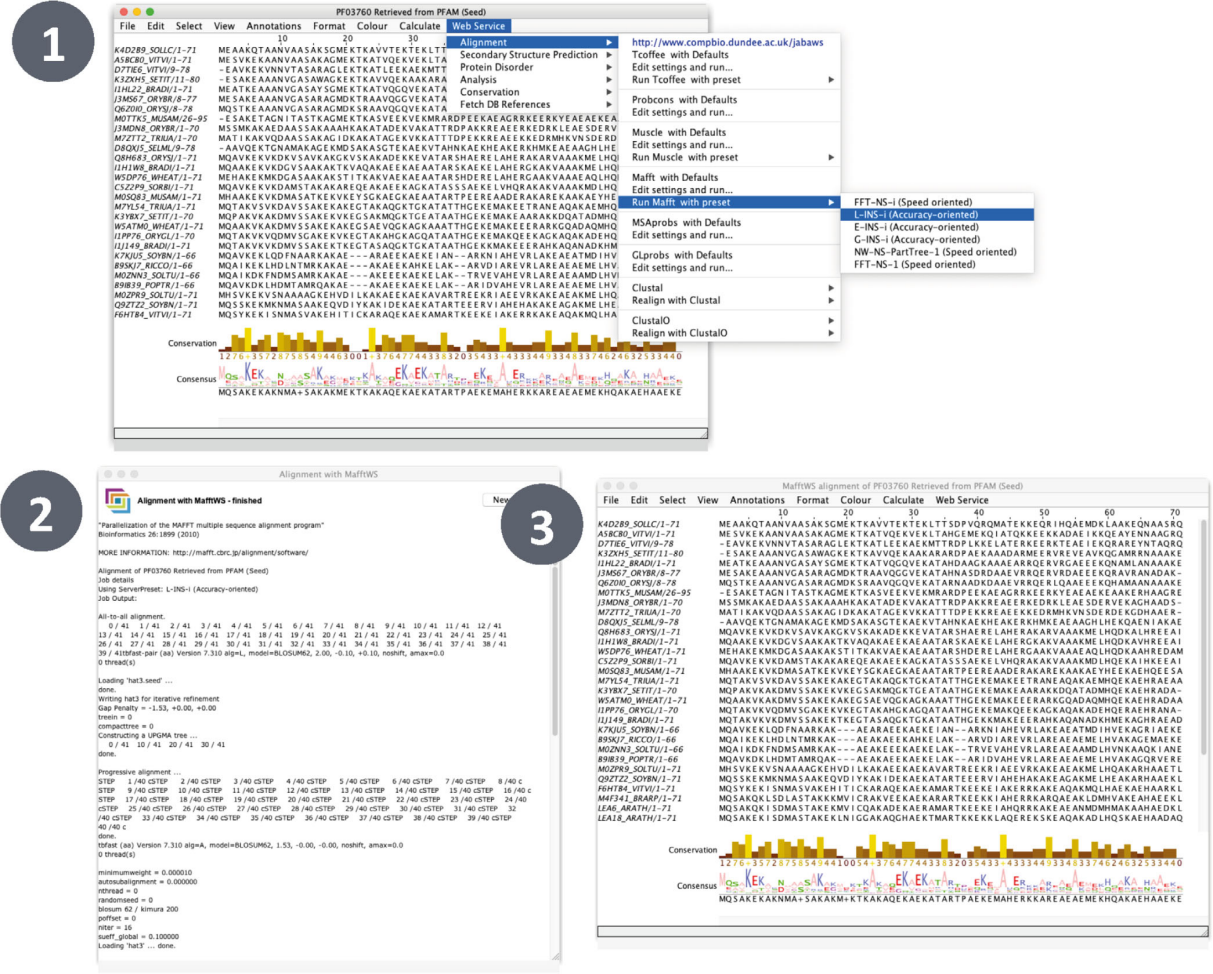
Running MAFFT^18^ L‐INS‐i alignment with Jalview's^1^ default JABAWS^10^ configuration. (1) Web Service → Alignment → Run Mafft with preset → L‐INS‐i. If custom parameters are desired they can be set in the dialog available through “Edit settings and run …” (2) A new window reports the job arguments and its progress. (3) The resulting alignment opens in a new window (n.b. the results MSA can be reopened with the “New Window” in the progress window)

Figure 2 illustrates how to run MAFFT^18^ on an alignment using the L‐INS‐i presets in Jalview. Jalview has a sophisticated yet intuitive interface to JABAWS. Jalview permits custom tool parameters, alignment, or realignment of alignment subsets and automatically displays results from JABAWS appropriately. In this example, the result is a new MSA and is displayed in a new alignment window. The JABAWS protein disorder or conservation tools create annotation tracks on the alignment on which they are run. Jalview also allows custom parameters to be set for a JABAWS tool via a dialog accessed under the appropriate *Web Service* submenus.

We have found the convenience of JABAWS beneficial in our own research. An analysis of all four disorder predictions in JABAWS in a set of known *O*‐linked β‐*N*‐acetylglucosamine transferase (OGT; 620 proteins) compared to a negative control set (1,164 proteins) showed that disorder was likely to be an element of OGT substrate recognition, despite the absence of clear sequence motifs.^30^ High‐throughput disorder predictions were tried as features in the prediction of 14‐3‐3 protein binding sites (see §2.4).^3^ JABAWS also simplified the calculation of conservation scores for several thousand Pfam^31^ alignments.^32^

### 2.2 *JPred4: A protein secondary structure prediction server*

The JPred4^2^ web server predicts secondary structure and solvent accessibility for a given protein sequence or multiple sequence alignment with the JNet 2.3.1 algorithm. A predicted protein secondary structure is useful in many ways when experimentally determined structures are unavailable. For example, secondary structure predictions can be used to improve multiple sequence alignments, as a starting point for 3D structure prediction, or to interpret patterns of conservation in an alignment.

Statistical and machine learning‐based approaches have proven effective at predicting protein secondary structure from sequence.^33^, ^34^, ^35^ JNet 2.3.1 has a secondary structure prediction three‐state accuracy (Q_3_; α‐helix, β‐strand, and coil) of 82.0%,^2^ which was as good as the PSIPRED^36^ and PredictProtein^37^ self‐reported blind test accuracies at the time of development. Since then, Xu and coworkers^38^ reported Q_3_ accuracies for JPred of 80–83% across a series of five other test datasets, values which were comparable to the other algorithms they tested and only slightly below the authors' DeepCNF‐SS program (82–85% across the five datasets).^38^ JPred4 solvent accessibility predictions are 90.0, 83.6, and 78.1% accurate for buried, part‐exposed, and surface residues, respectively.^2^

**Figure 3:**
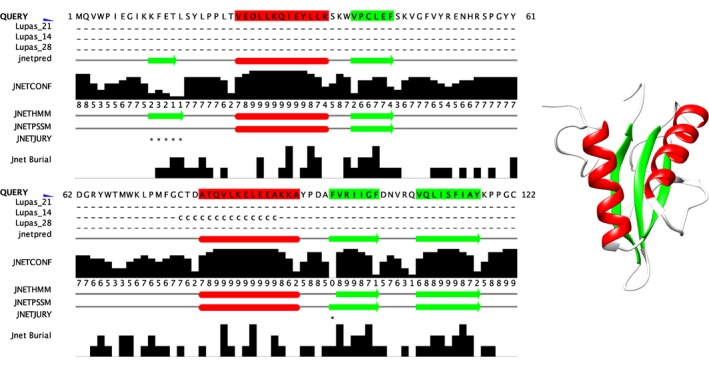
Illustration of a JPred4^2^ secondary structure prediction displayed in Jalview^1^ (left) and UCSF Chimera^40^ (right). Below the query sequence, JPred provides several annotation tracks for visualization in Jalview. These are the Lupas^39^ Coil predictions with varying window sizes (“‐“ = no coil; “c” = likely coil; “C” = coil); the final JNet prediction (red, helix; green, strand) followed by a confidence score for the prediction (0–9; least to highest confidence). These are followed by separate predictions where JNet is given only the profile HMM or PSSM and the JNETJURY track that indicates positions where these predictions differ (indicated by “*”). Finally, burial predictions are represented by a histogram of values ranging 0–3, representing no burial and burial at 25, 5, and 0% thresholds, respectively. The query sequence and structure illustration are derived from PDB ID: 3AXM^41^

JPred4 can make predictions for a single sequence, a batch of single sequences, or a precomputed multiple sequence alignment. The sequence pipeline begins by searching the PDB for homologues and will advise the user of any matches that are found since if the 3D structure of a homologue is known, this provides a strong guide to the secondary and tertiary structure of the protein and secondary structure prediction is less useful. The sequence is then checked against the DRSASP ProteoCache (see §3.2), and if found, the full JPred results are retrieved from the datastore within a few seconds. Otherwise, the sequence is queried against Uniref90 with PSI‐BLAST, and a nonredundant multiple sequence alignment is constructed from the matches. From here, JPred generates a profile HMM with HMMER and passes this and the PSSM from PSI‐BLAST to JNet and the Lupas coiled‐coil predictor.^39^ In the MSA pipeline, the profile HMM and PSSM are generated directly from the user‐supplied MSA, and these are fed to JNet without any PSI‐BLAST search. Figure 3 illustrates JPred results visualized with Jalview and UCSF Chimera. The JPred predicted secondary structure is shown in Jalview as an annotation track where green indicates strand and red indicates predicted helical regions. This coloring is then transferred to the mapped PDB structure 3axm^41^ through the Jalview‐UCSF Chimera interface to illustrate the accuracy of the prediction. JPred4 returns results in several formats: graphically by generating an SVG with Jalview; HTML formatted alignment with prediction tracks; PDF generated with Alscript^42^ and in Jalview^1^ via a JVL file (Jalview Launch file; requires Jalview ≥ 2.11 installed locally).

JPred4 can be accessed in multiple ways. The website provides a convenient interface to allow users to make secondary structure predictions for a single sequence, a batch of sequences, or for a user‐provided MSA. JPred4 predictions for a sequence or MSA can also be obtained from directly within Jalview. Alternatively, JPred4 can be accessed programmatically via its REST API, and a Perl command‐line client is available as the recommended interface. This allows users to submit, monitor, and retrieve JPred4 predictions *en masse* or as part of computational pipelines. The API client is a suitable means to obtain whole proteome scale JPred prediction sets without overloading the JPred4 server.

A good way to understand JPred's relevance is to see how others have applied JPred predictions to address problems. JPred can be applied in analyses involving a few proteins, whole proteomes, or other large sets of proteins or as part of new computational pipelines. An example of the application of JPred to guide experimental work is the identification of the paired amphipathic helix protein Sin3a interaction domain in the methylcytosine dioxygenases TET1 and TET3.^43^ The authors identified a common helical region in TET1 and TET3 outside of the known oxygenase and Zinc finger domains that was absent in TET2. The putative TET1–Sin3A interaction helix was confirmed experimentally with co‐immunoprecipitation, site‐directed mutagenesis, and NMR. JPred predictions were also used to assist the Cryo‐EM structure determination of the DNA‐bound PolD complex.^44^ High‐throughput applications of JPred include structurally rationalizing the distribution of aspirin mediated lysine acetylations in the human proteome;^45^ determining the factors affecting heterologous protein solubility^46^ and identifying kinases with a helix present in their activation loop across the human kinome.^47^ Lastly, JPred is an essential part of the QuanTest^48^ method for MSA benchmarking that compares MSAs containing sequences of known structure by assessing the accuracy of the JPred secondary structure predictions made from them.

### 2.3 *AACon*

**Figure 4:**
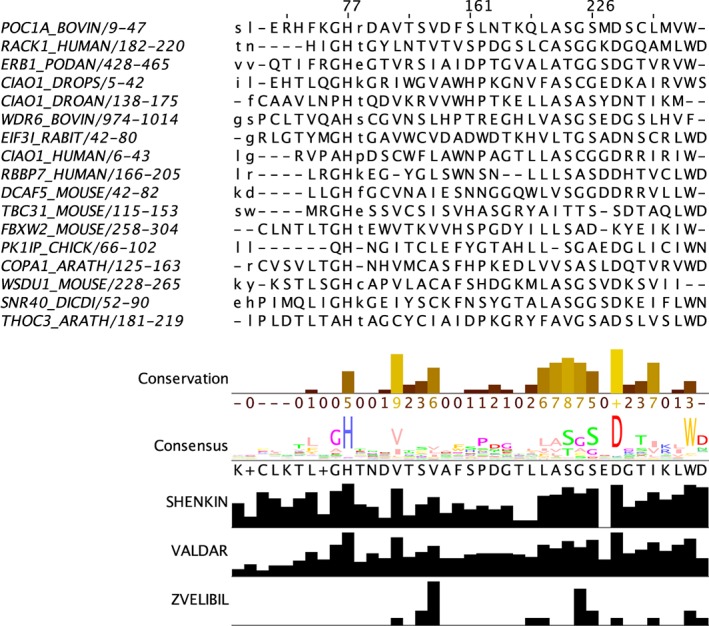
Comparison of evolutionary conservation scores. An excerpt of the Pfam^31^ WD40 repeat family (PF00400) is displayed together with Jalview^1^ annotation tracks representing five different conservation metrics (the scores were calculated for the first 89 SwissProt sequences in this Pfam, only the first 17 are shown). The Conservation and Consensus tracks are calculated by Jalview whilst the Valdar, Shenkin, and Zvelebil tracks are calculated with AACon via JABAWS called from the Jalview webservices menu

AACon is a Java implementation of 18 methods of scoring amino acid residue conservation in multiple sequence alignments. The majority of the methods are described in Valdar's 2002 review^49^ with additional algorithms that were developed in the Barton group. The methods include the symbol frequency‐based Shenkin score,^50^ the physicochemical property‐based Zvelebil score,^51^ the redundancy aware Valdar score,^52^ and the specificity‐sensitive SMERFS score.^4^ These examples illustrate how different scoring algorithms consider residue conservation as characterized by different features of the alignment. This point is demonstrated in a real‐world example in Figure 4, which compares five different conservation scores for an excerpt of the Pfam^31^ WD40 repeat family MSA. In this example, the scores do not all concur on what positions are most conserved in this alignment. Jalview's physicochemical conservation score highlights the consensus Asp and Val/Ile as the two most physicochemically conserved in contrast with the consensus, Valdar and Shenkin scores that all include the His and Trp consensus positions amongst the most conserved. Indeed, even the physicochemical‐based Zvelebil score identifies very different positions as the most conserved due to different treatments of gaps and aberrant or atypical residues.

AACon is accessible via the JABAWS^10^ web service, which as described §2.1, is available via Jalview or the JABAWS CLI client. AACon is also available as an executable JAR file, Java library, or its own web service. Users interested in analyzing conservation in only a few MSAs will probably find the Jalview–JABAWS interface sufficient for their needs. Studies that require high‐throughput conservation calculations or where a numerical comparison of different conservation scores is desired will best be served by either JABAWS Client or AACon executable. In this case, the user should determine whether remote execution would be advantageous and check if their alignments are within the Dundee JABAWS service sequence limits. The precise limits vary depending on what conservation scores are requested but range between 2,000 and 10,000 sequences of average length 1,000–10,000 residues depending on the requested scores; the precise limits can be queried with the JABAWS client. If these conditions are met, then the JABAWS Client is suitable; otherwise, it is recommended to use the AACon executable locally (https://github.com/bartongroup/aacon).

### 2.4 *14‐3‐3‐Pred*

14‐3‐3‐Pred^3^ is a webserver that predicts 14‐3‐3‐binding sites. 14‐3‐3 proteins regulate a variety of cellular processes by binding pairs of phosphorylated Ser/Thr residues on its target substrates.^53^ 14‐3‐3‐Pred combines predictions from PSSM, SVM, and ANN models, which were trained on a gold standard set of 14‐3‐3 binding sites created by a modest extension of the ANIA^54^ database and curated negative sequence set, into a consensus predictor. Recent applications of 14‐3‐3‐Pred include a screen of 106 putative substrates in tomato;^55^ the localization of the 14‐3‐3 target residues in the Nuclear receptor subfamily 1 group I member 2 protein^56^ and a target residue in the inactive tyrosine‐protein kinase transmembrane receptor ROR1.^57^

**Figure 5:**
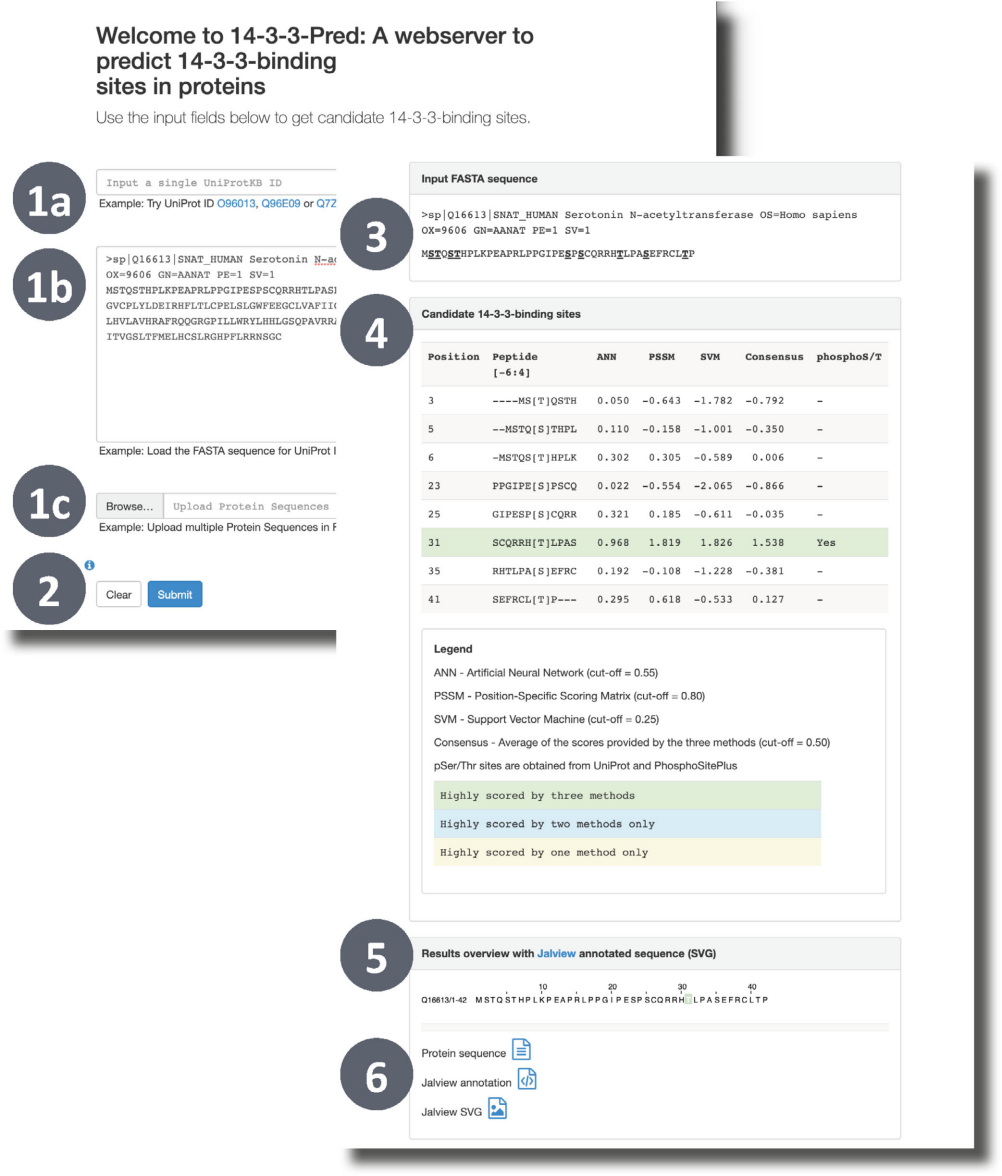
14‐3‐3‐Pred^3^ submission page (back). The website presents a form where you can enter either a UniProt accession (1a), a FASTA sequence (1b), or upload a set of sequences in a FASTA file (1c). The prediction is started by clicking “Submit” (2). 14‐3‐3‐Pred results page (front). The results indicate the query sequence with S/T sites highlighted (3); a table showing the query motifs, the prediction scores, and whether the site is known to be phosphorylated (4); a sequence view of the predictions (5) and download links including Jalview feature file format (6)

Figure 5 displays the 14‐3‐3‐Pred web interface where proteins of interest can be queried using single UniProt accession identifiers or as sequences in FASTA format. The results page displays a table with the site scores as well as information on the phosphorylation state of the respective Ser/Thr for each queried protein. Alternatively, a file containing up to 100 protein sequences in FASTA format can be uploaded. 14‐3‐3‐Pred then generates tabular results files that can be used to compare predictions, elaborate hypotheses, and prioritize laboratory experiments to investigate the predicted sites. Results can also be accessed programmatically using single UniProt IDs (“pid = <identifier>”) and specifying the output format (“out = <format>”) as JSON, CSV, or TSV. An example query is http://www.compbio.dundee.ac.uk/1433pred/pid=O96013&out=json.

**Figure 6:**
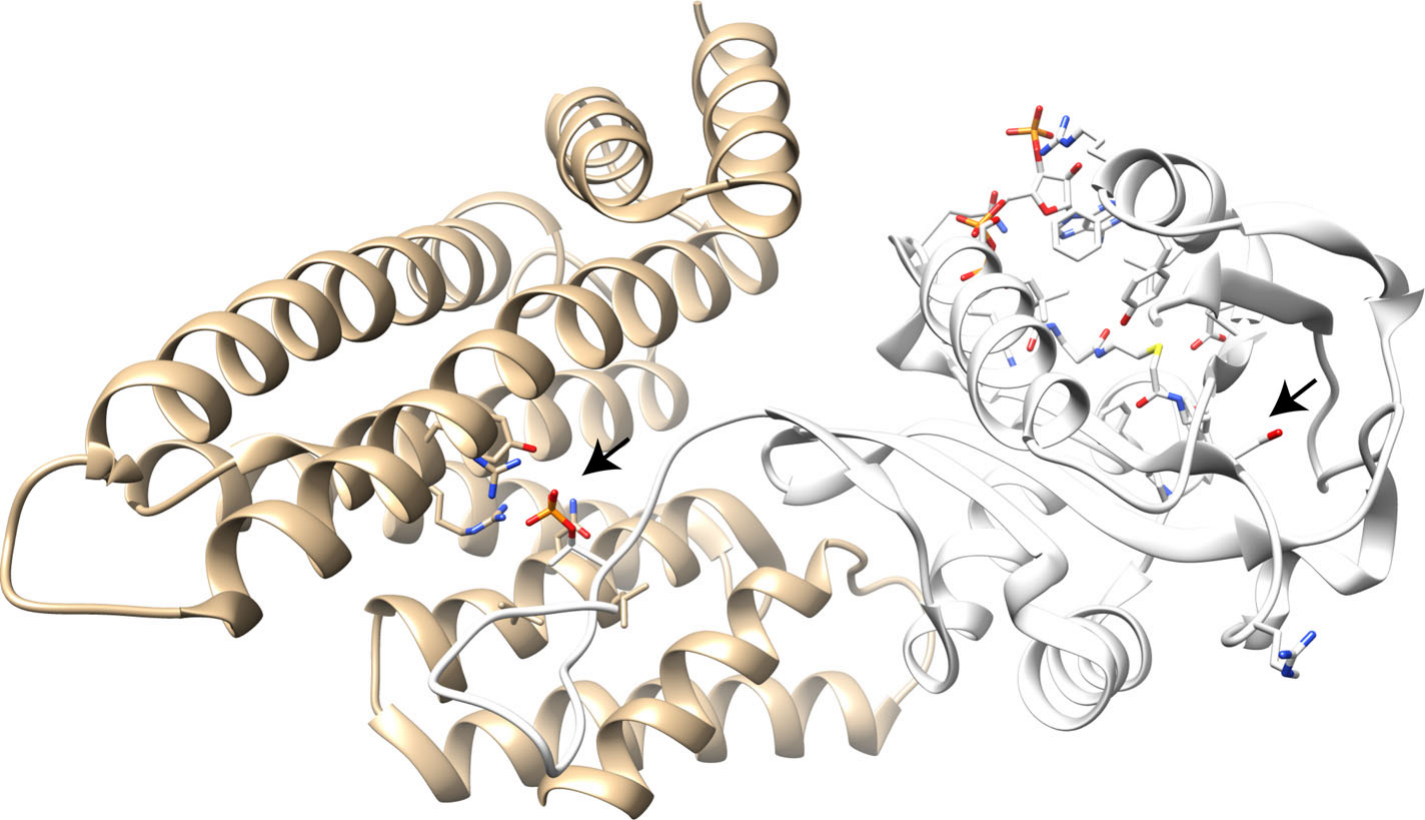
Illustration of Serotonin N‐acetyltransferase (right; white) in complex with 14‐3‐3 zeta (left; tan) showing the interaction of pThr31 with 14‐3‐3 zeta. The 14‐3‐3‐Pred predicted 14‐3‐3 targets pThr 31 and Ser 118 in Serotonin N‐acetyltransferase are indicated with black arrows. Figure adapted from PDB ID: 1ib1^58^ chains A and E, with UCSF Chimera and Jalview

Figure 6 illustrates the results of a 14‐3‐3‐Pred analysis on sheep serotonin *N*‐acetyltransferase. The prediction was run via the webserver, and the results downloaded as Jalview features. These were then loaded into Jalview, and Jalview's PDB lookup identified the structure 1ib1,^58^ and this was opened in UCSF Chimera via Jalview. Out of 22 Ser/Thr sites, 14‐3‐3 Pred correctly identifies pThr 31 as a 14‐3‐3 binding site with high confidence (i.e., all method concordance) whilst Ser 118 is falsely predicted to be a 14‐3‐3 binding site albeit with low confidence. A third high‐confidence positive prediction is found for pSer 205, which is not resolved in this structure.

### 2.5 *NoD*

NoD^9^, ^59^ is a predictor of nucleolar localization sequences (NoLSs) in proteins. NoLSs are short basic motifs that localize proteins to the nucleolus. The NoD algorithm is an artificial neural network (ANN) that was trained using three‐fold crossvalidation on 46 experimentally validated NoLs and negative sequences representing non‐NoL nuclear localization sequences and randomly selected nonnucleolar cytoplasmic and nucleoplasmic sequences. NoD predictions were computed for the human proteome, and 10 of the top scoring NoLSs were experimentally confirmed.^59^

**Figure 7:**
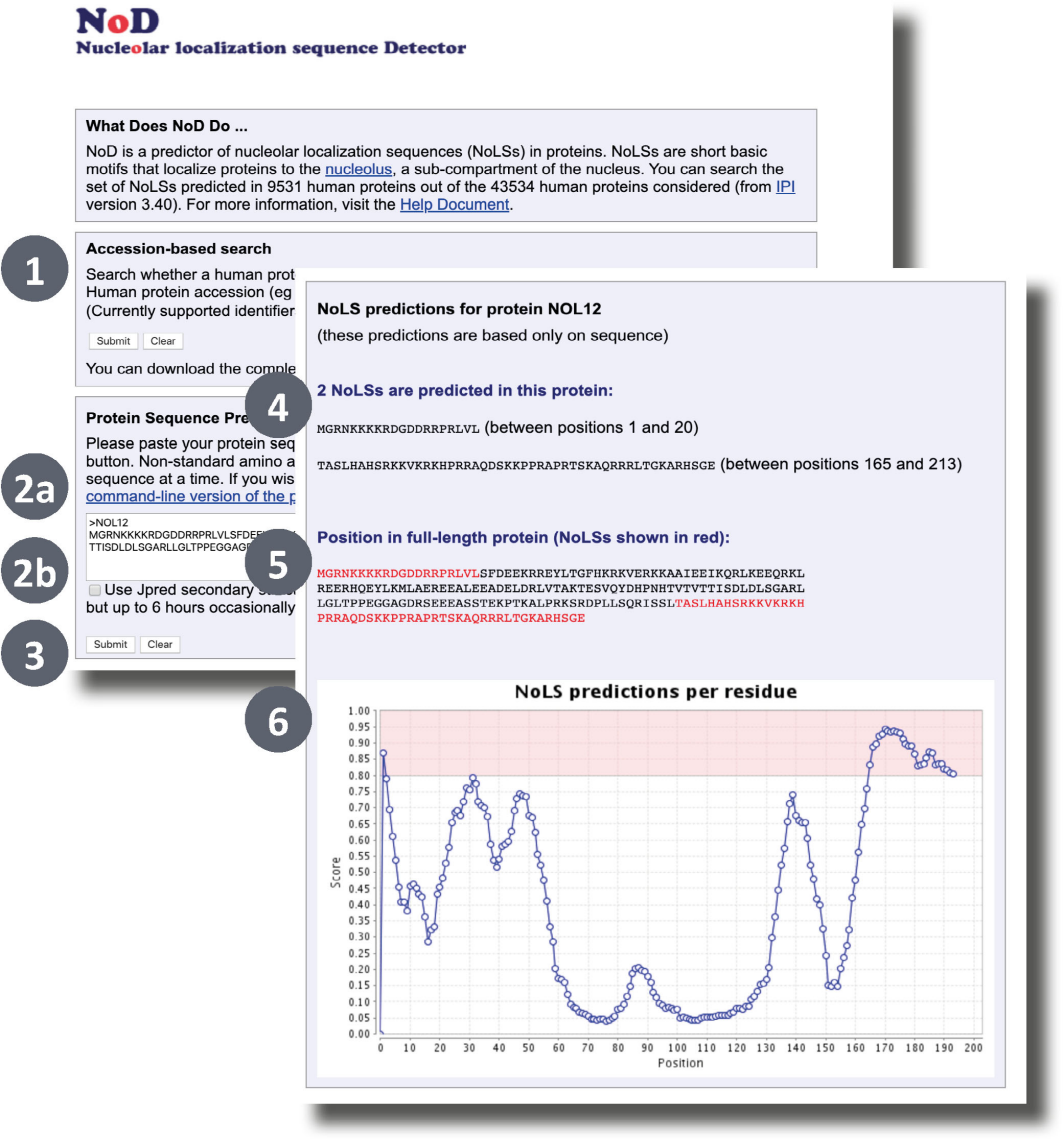
NoD^9^ input form (back). The user can input either a protein accession to query a precomputed set of results (1) or paste a FASTA sequence (2a) to run an ab initio prediction. If a sequence prediction is requested this can be done with or without using a JPred prediction as a feature (2b; n.b. NoD uses JPred3). The prediction is started by clicking “Submit” (3). NOD output form (front). Any predicted nucleolar localization sequences are shown both in isolation (4) and in context of the query sequence (5) and a line plot indicates the average score of 20 residue segments (6; see online help for more info)

Figure 7 illustrates the NoD submission and results pages. You can search the set of NoLSs predicted in 9,531 human proteins out of the 43,534 human proteins considered from IPI^60^ (version 3.40). NoLS predictions for an arbitrary protein sequence in FASTA format can be obtained via the text input box. If possible, full‐length protein sequences should be used to obtain maximum prediction accuracy. Optionally, users can decide to include JPred3^11^ secondary structure prediction in the prediction of NoLSs. This results in more accurate predictions but requires more computation time (usually around 10 min but up to 6 h is known). Once the protein sequence has been submitted, a waiting page is displayed providing users with a link to the output page. This link can be bookmarked and consulted later. The results page indicates the positions and sequences of any predicted NoLS. A graph of the predictor score along the length of the sequence is also shown. NoD can also be downloaded and run locally, in which case tabular output can be obtained more amenable to high‐throughput analyses.

The NoD server has been in continual use since its creation. A recent study employed NoD to scan for nucleolar localization motifs in Fbw7α, −β, and ‐γ isoforms.^61^ NoD correctly identified the nucleolar localization signal in Fbw7γ, suggested the presence of a weak signal for the nucleoplasmic Fbw7α, and reported no signal for the cytoplasmic Fbw7β. The NoLS in Fbw7γ was also shown to be the binding epitope for nucleophosmin (NPM1). Predicted NoLS in the CENP‐W and Tat proteins were also experimentally verified by the authors to bind NPM1.^61^ Predicted NoLs were subsequently found in p14arf, another NPM1 interactor.^62^ Mitrea et al.^63^ found that, 63% of a curated list of 83 NPM1 interactors had NoLS predicted by NoD, and many of these NoLs overlapped with the so‐called “multivalent R‐motifs” the authors hypothesized. In a separate study, Duan et al.^64^ used NoD to locate a suspected NoLS in the C‐terminal domain of poly(A)‐specific ribonuclease, which they then demonstrated experimentally was essential for nucleolar localization.

### 2.6 *Kinomer*

The Kinomer^5^, ^65^ webserver allows accurate identification of protein kinases (PKs) and their classification into kinase families. Kinomer also includes a browsable database of precomputed predictions of PKs in 43 eukaryotic genomes organized in kinase classes. Kinomer works by scanning sequences against a library of PK multilevel profile HMMs. The Kinomer profile HMM library comprises 38(+1) profile HMMs and is known as “KinaseLib2” (KL2). KL2 was developed by iteratively subdividing the known PK families by sequence similarity and testing the performance of profile HMMs built from these subgroups to recall and classify other known PKs. KL2 was determined to be more accurate than an alternatively trialled KinaseLib1 (KL1), which contained 12 profile HMMs, one for each of the eight known conventional eukaryotic protein kinase (ePK) and four atypical protein kinase (aPK) families. The ePKs are AGC, CAMK, CK1, CMGC, RGC, STE, TK, and TKL. The aPKs are Alpha, PIKK, PDHK, and RIO. The Kinomer database was built by scanning whole proteomes against the KL2 multilevel profile HMM library. Recent applications include the classification of kinases in the fungal pathogen *Cryptococcus neoformans*.^66^ They compared the proportions of kinase classes in the fungal pathogens, *C. neoformans*, *Candida albicans*, and *Aspergillus fumigatus*. The *C. neoformans* Kinase Phenome Database contains Kinomer annotations.

**Figure 8:**
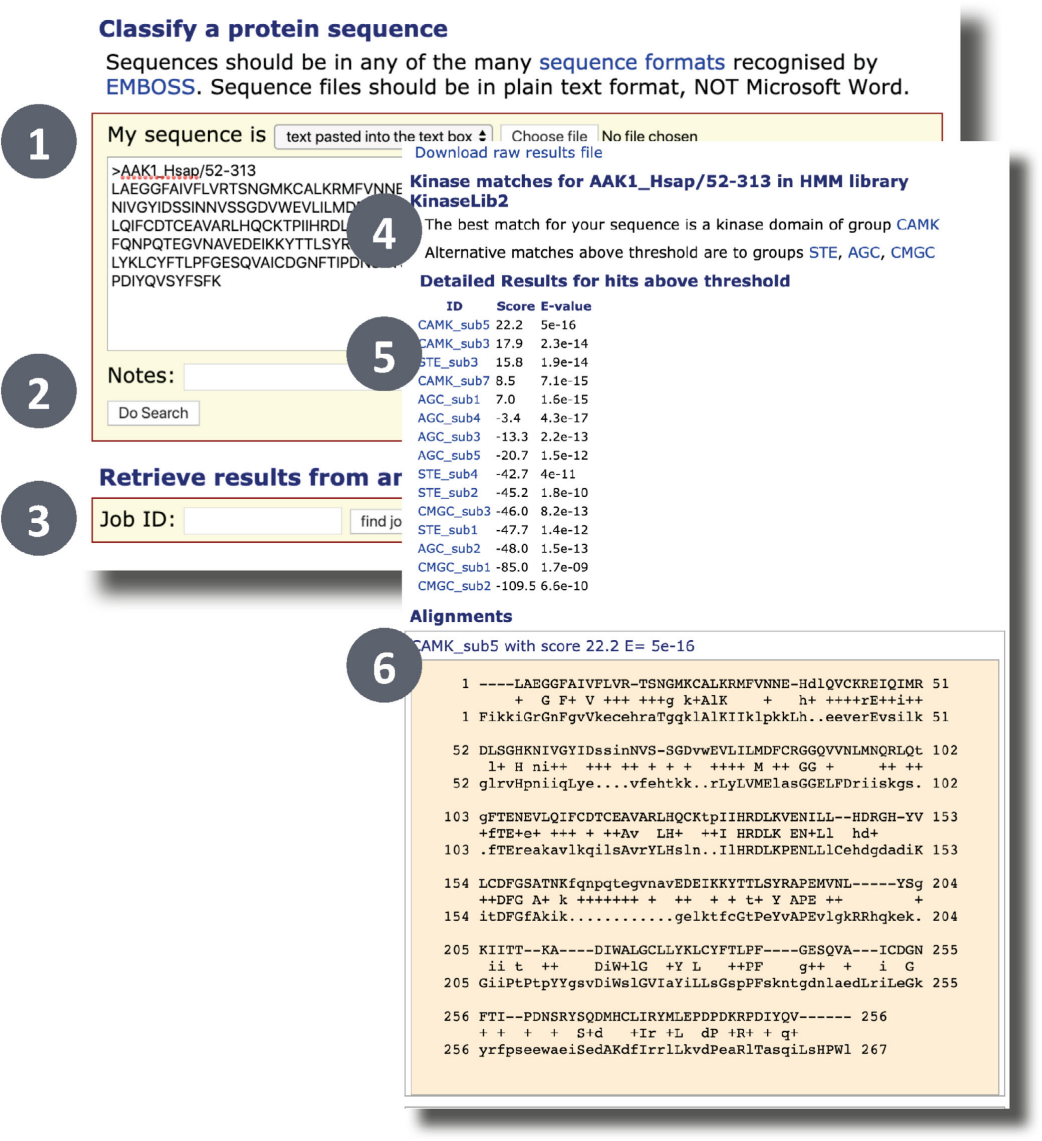
The Kinomer^5^ search input (back) and output forms (front). The user can paste a FASTA sequence (1) and start the classification by clicking “Submit” (2) or retrieve the results from a previously submitted job using the Kinomer job ID (3). If there are any hits to the Kinomer profile HMM library above Kinomer's thresholds, then the best matching kinase group (4) and alternative matches are reported (5). Alignments for each hit are shown below (6) and can be downloaded from the top of the page

Users can either browse the Kinomer database or classify a sequence by scanning against the Kinomer profile HMMs. Figure 8 displays the Kinomer sequence classification submission page. From here, a single sequence can be input via the text box or uploaded in FASTA format. The results of previous jobs can also be retrieved via the job ID. The Kinomer results page reports the best classification for the input sequence along with high‐scoring alternative matches. Scores for all potential matches are also shown as well as the alignments corresponding to each match.

### 2.7 *Xtal*

Xtal^6^, ^7^, ^8^ is a collection of methods that predict the likelihood of a protein succeeding in a crystallization experiment. Predicting the crystallization propensity is useful for construct design and prioritizing targets for structural genomics projects. The algorithms within Xtal are the OB‐Score,^6^ ParCrys,^7^ and XANNPred.^8^ The Xtal algorithms were developed over several years, and each represented an improvement over the previous in terms of predictive performance as a result of improved algorithms and training data. Despite the precedence of XANNpred, which in our hands is the most accurate of the three, we provide and maintain the OB‐Score and ParCrys since they remain useful and display their own strengths. For example, although it was our first crystallization propensity predictor, OB‐score was one of four algorithms determined to be ideal for fast proteome‐wide target selection in a recent review.^67^

The OB‐Score^6^ predicts whether a protein is likely to lead to a successful structure determination by calculating and assessing its predicted isoelectric point (p*I*) and grand average of hydrophobicity (GRAVY).^6^ This is achieved by comparing the p*I* and GRAVY values to proteins that have been successfully crystallized. This relatively simple approach yielded an accuracy of 69.8% with AUC 0.711 on an independent test dataset.^7^ The OB‐Score was calculated for nearly 250 proteomes to compare each organism's suitability for high‐throughput crystallography as well as the sequences in Pfam 17.0^68^ to identify a good candidate template structure for the protein families. These datasets remain available for download from the website for archival reasons, but a researcher wishing to conduct a similar analysis is urged to use a recent dataset. For this reason, we recently calculated OB‐Scores for 30,498,342 sequences across 16,449 families from Pfam 31.0; this new dataset and future updates can be found at http://www.compbio.dundee.ac.uk/xtal/ob_datasets/. It is also simple to calculate OB‐Scores on a large scale via the distributed Perl application, for example, it took less than 30 s to calculate OB‐Scores for the 42,500 sequences in PF00001.20. The OB‐Score webserver returns the raw value of the OB‐Score. This is interpreted with the following thresholds: a predictive threshold of 0.809 optimized accuracy over the test dataset; OB‐Score ≥ 5 can be considered high scoring, and 1.5 yields an optimal MCC (Matthews' Correlation Coefficient^69^) on a real‐world dataset. The OB‐Score was also recently employed to prioritize tractable targets for insecticides against the malaria vector *Anopheles gambiae*.^70^

ParCrys^7^ is a Parzen Window‐based estimator of crystallization propensity that uses p*I*, hydrophobicity, and the frequencies of S, C, G, F, Y, M residues only. The sequence is predicted as one of three classes: difficult to crystallize (“recalcitrant”); amenable to crystallization (“amenable”); or very amenable to crystallization (“high‐scoring”). Extensive feature selection was performed during the development of ParCrys. ParCrys surpassed the OB‐Score even when using a reduced feature set of only p*I* and hydrophobicity, indicating that the Parzen Window model itself provided significant advantages. The inclusion of the remaining residue frequency features led to further performance gains compared to the OB‐score. Adding other amino acids as features besides S, C, G, F, Y, and M led to performance degradation, which was reasoned to be due to correlation between p*I* and charged residue frequencies and consequently a no‐benefit decline in the parameter/observation ratio. ParCrys achieved an accuracy of 79.1% with AUC 0.844.

**Figure 9:**
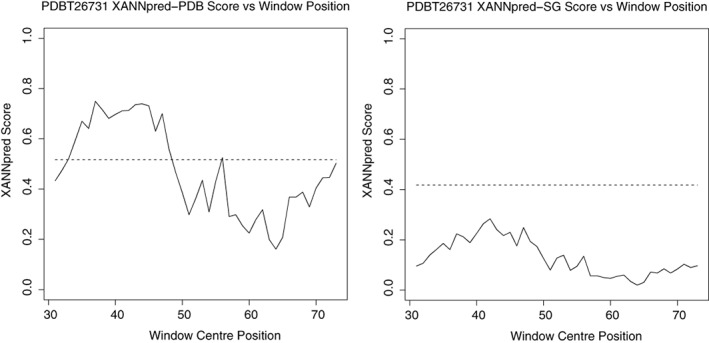
XANNpred^8^ windowed predictions for XANNpred‐PDB (left) and XANNpred‐SG (right). The prediction threshold is indicated by the dashed line (0.517 for XANNpred‐PDB; 0.418 for XANNpred‐SG). The windows are 61 residues long and so the first window is centered at residue 31. A relaxed interpretation considers high‐scoring regions as those residues that are contained within a high‐scoring window (i.e., ±31 residues of the window center). A conservative interpretation is restricted to where the window centers are above the prediction threshold. XANNpred provides these figures as attachments in the results email

XANNpred‐PDB and XANNpred‐SG (together XANNpred^8^) are neural networks that predict whether a protein is likely to produce diffraction quality crystals based on amino acid frequencies (including dipeptides), sequence length, and molecular weight as well as predicted p*I*, hydrophobicity (GES), secondary structure (JPred), transmembrane regions (TMHMM2), and protein disorder (RONN).^8^ The two neural networks differ only in their training where XANNpred‐PDB was trained with a positive training set derived from the PDB and XANNpred‐SG's positive training set was derived from the now retired PepcDB, which included sequences that were known to crystallize but had not necessarily been solved at the time. XANNpred achieved AUC 0.854.^8^ The XANNpred webserver calculates the required sequence features and runs both neural networks to provide the prediction results. XANNpred also provides predictions for subsequences within the query via a sliding window approach. This provides region‐specific crystallization propensities that are particularly useful for construct design. Figure 9 illustrates how the XANNpred windowed predictions vary over the XANNpred demo sequence (PDBT26731). In this example, the windows centered on residues 33–47 are above the threshold for XANNpred‐PDB; this suggests that residues 2–78 are more amenable to crystallization than the remaining sequence (i.e., these residues are in at least one high‐scoring 31 residue window).

**Figure 10:**
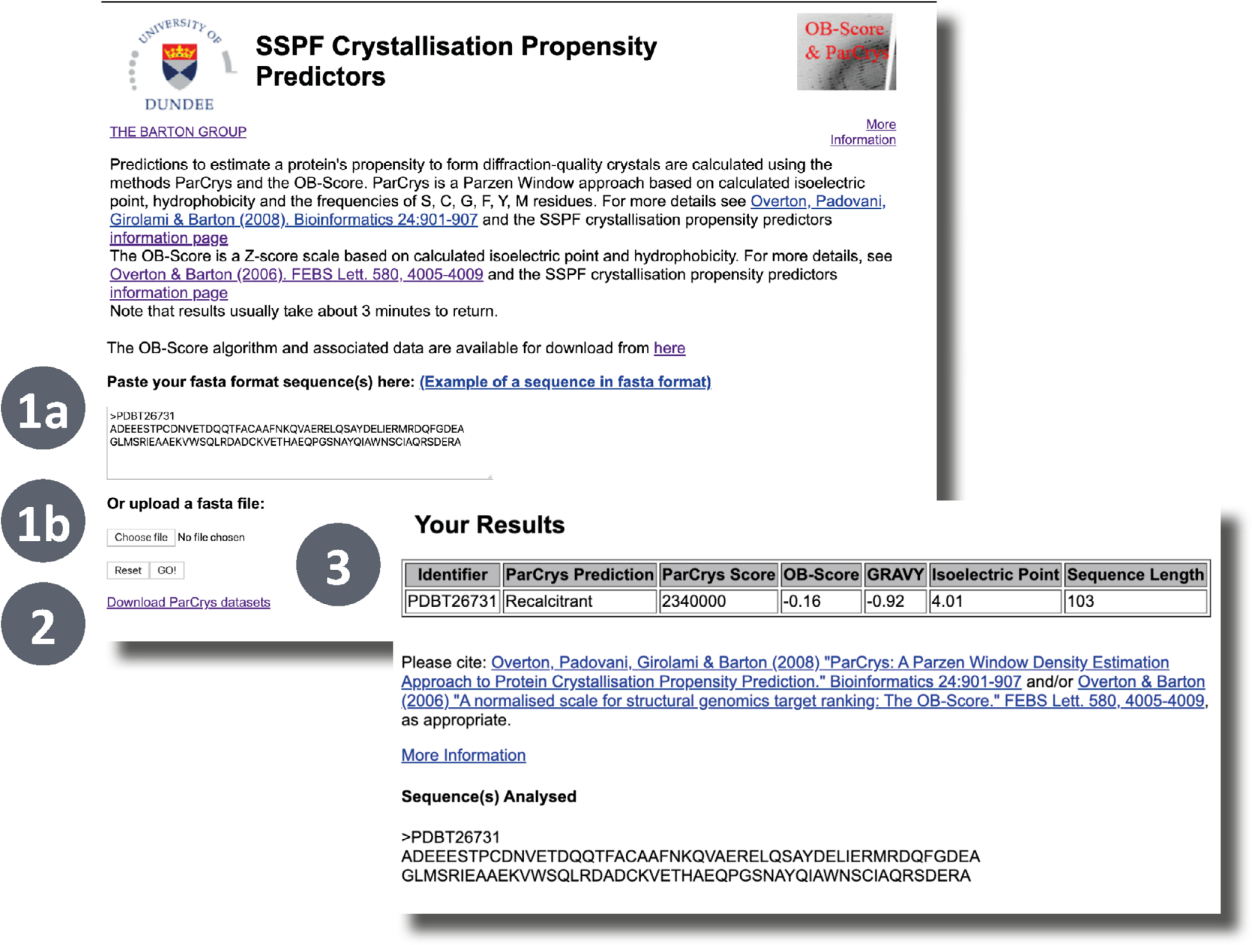
XTal input form for OB‐Score^6^ and ParCrys.^7^ Xtal output form. Users can input a sequence or multiple sequences by pasting FASTA format into the textbox (1a) or uploading a FASTA file (1b). The prediction is then run by clicking the “GO!” button. A link to download the ParCrys datasets is provided at the bottom of the page. Once the calculation is complete, the results page will load and display a table listing the OB‐Score and the ParCrys score and prediction for each submitted sequence alongside the GRAVY, pI, and the sequence length

All three predictors in Xtal are available via web forms. The OB‐Score^6^ and ParCrys^7^ are accessed via a single submission page whilst XANNpred^8^ submissions are made via its own page. Figure 10 illustrates the OB‐Score/ParCrys submission and example results page (n.b. the submission form for XANNpred is very similar). A user can submit sequences in FASTA format via a text box or file upload. After a few moments, OB‐Score and ParCrys predictions are reported via a results page in an HTML table. XANNpred predictions are returned in tabular format via email. If requested, XANNpred windowed predictions are included in the results email as PDF attachments (Figure 9). Alternatively, the OB‐Score Perl application and data can be downloaded and run locally after following some minor configuration instructions returning results in TSV format (Tab Separated Values). Precalculated OB‐Scores are available for Pfam 31.0.

### 2.8 *AMAS*

AMAS^14^ is an hierarchical conservation analysis algorithm based on a set representation of amino acid physicochemical properties. The AMAS server has been in operation since 1994. In addition to the standard identification of residues that are conserved in all sequences at a position, AMAS can indicate various types of subgroup conservation. For instance, the AMAS output differentiates columns that are conserved in some but not all subgroups (*conserved and similar*; e.g., where a structural constraint is lost in particular subgroups) from columns that are conserved in most subgroups but where each subgroup conserves a different feature (*conserved but different*; e.g., sites important for specificity). This description is admittedly abstract, and a more complete illustration can be found in the AMAS paper.

**Figure 11:**
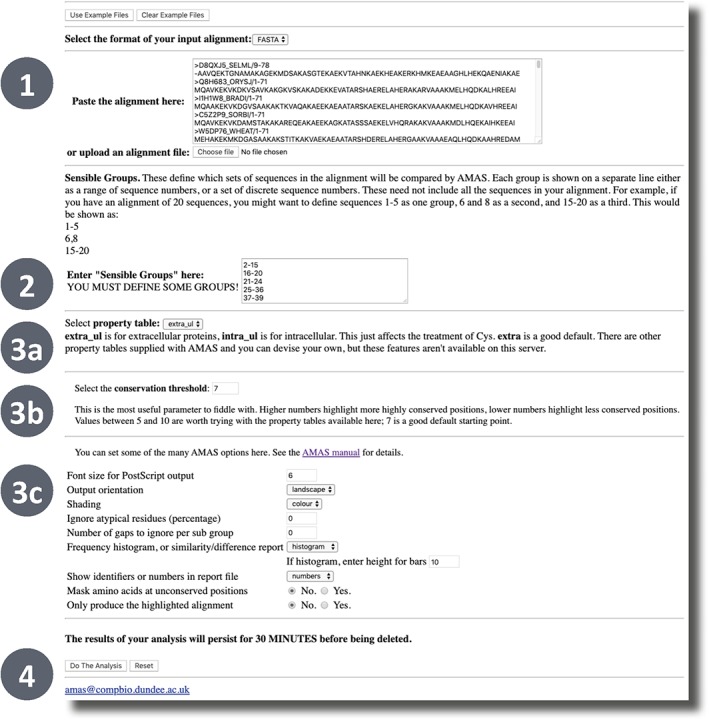
The AMAS^14^ input form. AMAS accepts FASTA, AMPS or Pfam formatted alignments via the textbox or file upload (1). Groups are defined via a textbox with one line per group and sequences referred to by their row index (2; e.g., “1–5” on a line defines a group of the first five sequences). The job can then be started with default parameters by clicking the “Do The Analysis” button (4) or advanced options may be set. These include the property table (3a), the conservation threshold (3b) and other formatting and analysis options (3c)

Figure 11 displays the AMAS submission page where users can run the analysis on their own multiple sequence alignments. FASTA, PFAM, or AMPS formatted alignments may be pasted directly into the provided textbox or uploaded from the user's local storage. AMAS also requires the user to provide subgroup classifications. Suitable groups could be derived from overall sequence similarity, functional similarity, or taxonomic relationships. Group membership is indicated by lines of comma delimited sequence indexes or ranges as indicated in the paragraph preceding the textbox. Note that the AMAS conservation analysis can be run with only a single group specified but, in this case, only the standard conservation score can be returned. The AMAS analysis can then be run with default settings by clicking the “Do The Analysis” button at the bottom of the page.

**Figure 12:**
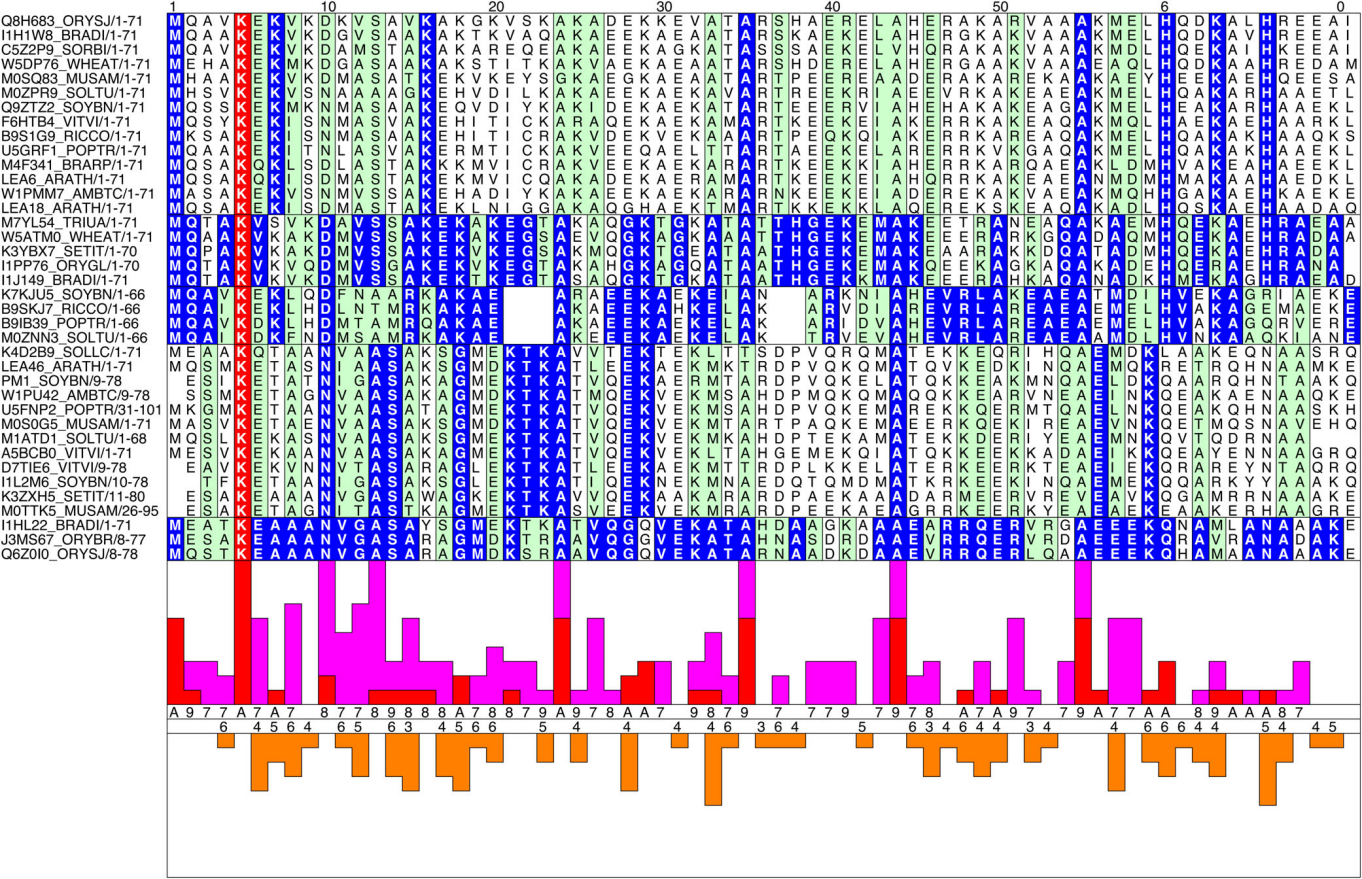
AMAS^14^ results visualization of an illustrative analysis upon Pfam PF03760. The alignment illustrates within group and between group conservation. Within group conservation is illustrated by block shading within the subgroups: blue indicates subgroup identity whilst green indicates property conservation. Additionally, red shading indicates total conservation across all groups. The histogram displays the similarities (orange) and differences scores (violet). The visualization is generated with Alscript.^42^ AMAS, Analysis of Multiply Aligned Sequences

Figure 12 illustrates the AMAS output visualization. The block coloring indicates the subgroup conservation, distinguishing identity in all subgroups (red), identity within a subgroup (blue), and conservation within a subgroup (green). The histograms summarize the AMAS comparison of the subgroups. The upper histograms show the overall conservation (red) and subgroup similarities (pink) whilst the lower histogram (orange) shows the average of the subgroup differences. The most dissimilar sites in terms of subgroup–subgroup comparison (i.e., large values on the subgroup differences histogram) are most likely to be important for specificity and are worth closer inspection. AMAS results are also available in text format.

Several important settings can be adjusted. The *property table* selection defines the amino acid physicochemical set memberships used to define the properties that can be conserved. Three options are available via the web interface: *extra_ul* is recommended for extracellular proteins where Cys is assumed to form disulfide bonds whilst *intra_ul* is recommended for intracellular proteins where Cys are assumed to be present as free thiols. The third option available *ch* is specifically for detecting conserved charges and changes in the polarity of conserved charges in certain subgroups and defines positive (His, Arg, and Lys), negative (Glu and Asp), and charged (His, Arg, Lys, Glu, and Asp) sets. The *conservation threshold*, *T*, defines what AMAS will consider to be a conserved position in a subgroup or subgroup pair. Higher (*T*) values will also result in a more specific analysis since only subgroup pairs where both subgroups have individual conservation scores > *T* are evaluated. Note that *T* must be less than the maximum possible conservation score (*C*_max_), which is determined by the number of properties in the property table; the server will error and report the allowed values if this rule is broken. The parameters labeled “Ignore atypical residues” and “Number of gaps to ignore per sub group” influence how sensitive the conservation score is to gaps and potentially aberrant residues. The remaining parameters in the lower options section control the formatting of the Alscript^42^ output alignment. Of particular note is the “Frequency histogram, or similarity/difference report” option, which controls whether summaries of the subgroup pairwise comparisons are shown (*histogram*; best when there are many subgroups) or the individual subgroup pair conservations are shown (*differences*; clearest when there are only a few groups).

## 3 NEW SERVICES UNDER DEVELOPMENT

We are currently developing several new services for DRSASP. Slivka is an evolution of the JABAWS^10^ concept written in Python that is designed to improve upon JABAWS' limitations. ProteoCache is the DRSASP “data warehouse,” at its core it is an Apache Cassandra database designed to hold and return precalculated results for all DRSASP tools, accelerating performance and providing a means to perform integrated analyses across our resources. ProteoFAV, ProIntVar, and VarAlign are Python packages that we created to meet our own research requirements for carrying out integrated analyses across protein sequences, multiple sequence alignments, 3D structures, and human genetic variation. These tools are discussed individually in brief below; further detailed discussion of their capabilities will be published upon each tool's release.

### 3.1 *Slivka*

Slivka is a new web service framework currently in development that will supersede the JABAWS framework. Slivka is implemented in Python with Flask, ZeroMQ, and MongoDB. Key advantages of Slivka compared to JABAWS are significantly simplified tool configuration, better facilities for tool chaining, and the capability of Slivka to generate tool specific‐web forms. Tool configuration in Slivka requires just two YAML files: a run configuration file to specify the command‐line interface of the tool and a form configuration that specifies the parameters exposed through the web API. Files uploaded to or generated by (i.e., results) the Slivka server for analysis (e.g., sequence files, MSAs) can be referenced via a *uuid*, which facilitates tool chaining since results can be referenced server side. Slivka is currently in advanced testing stages, and we expect to deploy a public production server early in 2020.

### 3.2 *ProteoCache*

ProteoCache is a database containing precomputed results of DRSASP and other applications for whole proteomes built with Apache Cassandra together with a Node.js API based on DataStax's cassandra‐driver. Apache Cassandra is a scalable and robust NoSQL database. At the time of writing, the database contains JPred4 predictions (including full alignments and PSI‐BLAST profiles) for most of the Human (57,823 sequences; 78%), *S. cerevisiae* S288C (5,049; 83%), and *E. coli* K‐12 (4,144; 94%) UniProt reference proteomes as well as disorder predictions for 79,513 sequences from the four disorder predictors provided by JABAWS. Tables in ProteoCache are indexed by sequence to allow fast lookup of new DRSASP queries. Currently, JPred4 interfaces with ProteoCache to improve the performance of JPred4 for previously run sequences. Our goal is that all DRSASP applications will similarly interface with the ProteoCache to improve performance of our web services. The ProteoCache itself will in the future be able to serve bulk downloads of whole proteomes or other large selections of sequences and also permit complex queries over the data.

### 3.3 *ProteoFAV, ProIntVar, and VarAlign*

Over the last few years, we have been researching how human genetic variants are distributed in proteins with respect to protein structure and conservation.^32^ This has led to the development of software that simplifies the complex task of connecting the heterogeneous data derived from variants, protein sequences, protein structures, and multiple sequence alignments. Our approach is to represent the data as Pandas DataFrames. Once all these data are harmonized, we can conduct complex queries and aggregations. For example, “return all missense variants at residues where the position is conserved and involved in a hydrogen bond with a ligand in Pfam PF00001” and “count all missense variants in each alignment column of PF00017”. This software is being developed as a series of Python modules, and we will release the libraries and provide a web service through Slivka upon journal publication of an updated version of our analysis of human variation in Pfam alignments.^32^

**Figure 13:**
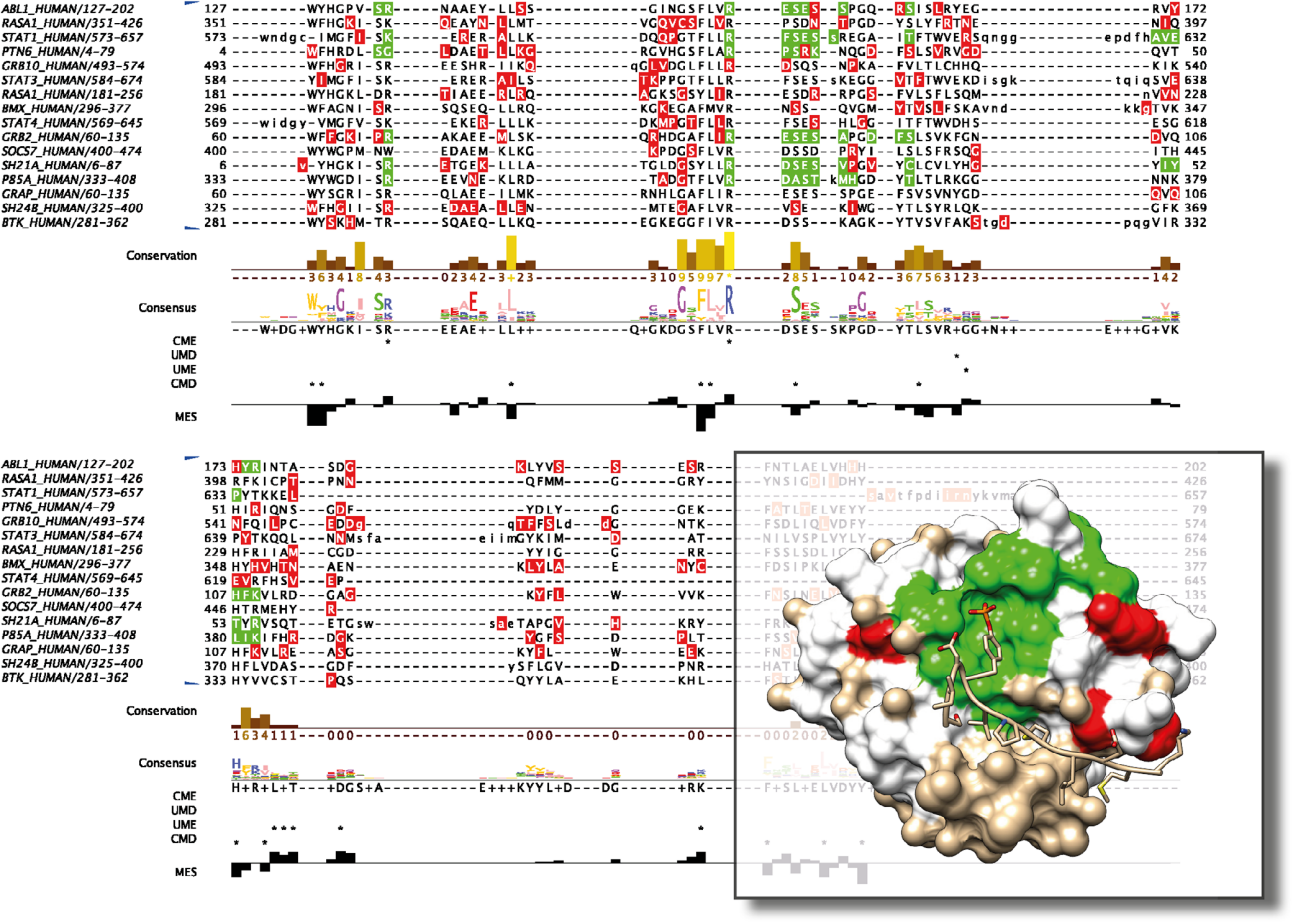
Example output from VarAlign and ProIntVar analysis^32^ of SH2 domains from Pfam^31^ PF00017 visualized with Jalvew^1^ and UCSF Chimera.^40^ In the alignment, nine of the most missense depleted SH2 domains are shown. The locations of missense variants from the gnomAD^71^ dataset are shown as semitransparent red features. The locations of residue–ligand interactions by ligands that bind in the SH2 canonical binding site are shown in semitransparent green. In these proteins, no missense variants occur at these positions (i.e., these features do not overlap). Four annotation tracks are shown, from top to bottom: Jalview calculated consensus; whether positions are classified as unconserved‐missense depleted (UMD), unconserved‐missense enriched (UME), conserved‐missense enriched (CME), or conserved‐missense depleted (CMD).^32^ The structure shows the interaction between the SH2 domain of phosphatidylinositol 3‐kinase regulatory subunit alpha and the platelet‐derived growth factor receptor beta phosphotyrosyl peptide in PDB ID: 2IUI. The locations of missense variants from the gnomAD dataset are shown in red. The locations of residue–ligand interactions by ligands that bind in the SH2 canonical binding site—in any structure that maps to this protein—are shown in green

Figure 13 illustrates one view of these data in Jalview. gnomAD^71^ variants were mapped to the residues in the Pfam^31^ SH2 domain alignment and are formatted as Jalview features with VarAlign. The sequences shown in Figure 13 are among the most missense depleted (constrained) human sequences in this family and were identified in Jalview by *View* → *Feature settings…* → *Sequence sort by Density* when only the missense variants were shown. VarAlign also fetched protein–ligand interaction data for all sequences in the alignment from the PDBe with ProIntVar. Rendering these data as features in Jalview allows the identification of colocated missense variants at these sites if there are any. Jalview allows quick visualization of these features on a mapped protein structure through its integration with UCSF Chimera.

### 3.4 *DRSASP workflows in Jalview*

Jalview^1^—a program for multiple sequence alignment editing, visualization and analysis—provides an interface to many of the DRSASP tools. This enables users to carry out sophisticated workflows that combine DRSASP tools and Jalview's built‐in analysis capabilities interactively. For example, a useful Jalview workflow is to cluster sequences iteratively, prune outliers, and align the remaining sequences. Once the alignment is judged sufficiently accurate, further DRSASP services can be invoked to calculate residue conservation, predict specificity‐determining sites (SMERFS; *Calculate* → *Calculate Tree* → AMAS), and predict structural features (solvent accessibility, secondary structure, and disorder). This rich annotation set can help interpret experimental observations (e.g., UniProt mutation data) and/or provide an enhanced understanding of the protein by projecting them onto structure in a Jalview linked MSA‐structure session. Other examples have been provided in the preceding sections.

## 4 GENERAL DEVELOPMENTS

We are committed to improving our software and data practices by working towards implementing OSS recommendations^72^ and the FAIR principles.^73^ In this vein, we will continue to add DRSASP resources to the bio.tools registry,^74^ deposit annotations in collaborative repositories (e.g., PDBe‐KB, see below), and make datasets and code publicly available. Some DRSASP projects align well with these ideas in their very concept. For instance, the JABAWS and Slivka frameworks will enhance the interoperability (aggregate services) and reproducibility (consistent execution environment) of bioinformatics tools in general whilst the ProteoCache promotes data reuse and integration.

A relevant development is our work to improve DRSASP's efficiency in whole proteome analyses. The precomputed data in ProteoCache (§3.2) is one aspect of this. Another direction we have pursued is the annotation of PDB structures in collaboration with the PDBe‐KB^75^ project as a data depositor. So far, we have annotated the set of human sequences in PDBe with 14‐3‐3‐Pred predictions. This resulted in 1,941 representative PDB chains receiving at least one positive prediction. These are accessible via PDBe‐KB (e.g., https://www.ebi.ac.uk/pdbe/pdbe‐kb/proteins/Q92879; see expanded “Predicted PTM sites” track in “Functional Annotations” section). Python scripts to assist running DRSASP tools in a high‐throughput manner and generating PDBe‐KB compliant JSON output are available from https://github.com/bartongroup/FM_FunPDBe.git.

As part of the PDBe‐KB deposition process, we were required to conform DRSASP results data to an agreed upon JSON standard. This effort is the beginning of a larger effort to harmonize the data out from DRSASP tools. A further step will be to ensure this work is efficiently translated into better Jalview integration for the DRSASP tools that are not currently well integrated. This might be achieved by introducing PDBe‐KB JSON parsing to Jalview or by converting the JSON to an existing Jalview format. Whilst this does not constitute full Jalview integration (i.e., the services are not called from Jalview), this may prove a useful stopgap and is worthwhile anyway to enable high‐throughput data generation where it is advantageous to generate required data in bulk (e.g., an analysis on Pfam might generate Jalview compatible annotations for 1000s of alignments, these annotated families can then be “browsed” with Jalview).

Lastly, we are making improvements in the testing and portability of DRSASP tools. A key priority in the short term is to improve the deployment of the DRSASP tools with the initial focus being JPred. This will involve applying modern technologies such as containerization (e.g., Docker) or modern dependency management solutions (e.g., Conda). In addition to simplifying our internal maintenance workflows, this will have the added advantage of simplifying the local installation of JPred so that users will have the option of running a local instance. Moreover, improving the portability of our software is an important component of our efforts to ensure our work is as reproducible as possible. On the technical front, we have also made improvements to DRSASP service reliability through the introduction of continuous monitoring. In addition to standard HTTP checks, we now use end‐to‐end interface tests for JPred and JABAWS services.

## 5 CONCLUSION

The DRSASP provides several bioinformatics web services for the scientific community. The tools address a wide variety of biological questions but are connected by the common themes of protein sequence analysis and structure prediction. The services provide secondary structure prediction, disorder prediction, multiple sequence alignment, functional site prediction, and more. DRSASP tools are accessible via web forms, programmatic APIs, and some are suitable for local installation. A unique aspect of DRSASP is its tight integration with Jalview.

As well as maintaining and continually developing existing tools, DRSASP has several new services that are close to release. Slivka and ProteoCache will improve the delivery of DRSASP services but they will also enable new developments in the future. (e.g., aggregated services and large‐scale integrated analyses). ProteoFAV, ProIntVar, and VarAlign are new services close to release that will enable new research, especially at the intersection of human genetics and protein structure.
